# GPR37 Receptors and Megalencephalic Leukoencephalopathy with Subcortical Cysts

**DOI:** 10.3390/ijms23105528

**Published:** 2022-05-16

**Authors:** Adrià Pla-Casillanis, Laura Ferigle, Marta Alonso-Gardón, Efren Xicoy-Espaulella, Ekaitz Errasti-Murugarren, Daniela Marazziti, Raúl Estévez

**Affiliations:** 1 Unitat de Fisiologia, Departament de Ciències Fisiològiques, Genes Disease and Therapy Program IDIBELL-Institute of Neurosciences, Universitat de Barcelona, L’Hospitalet de Llobregat, 08907 Barcelona, Spain; adria.pla9@gmail.com (A.P.-C.); laura.ferigle@ub.edu (L.F.); malonsga@gmail.com (M.A.-G.); efrenxicoyespaulella@gmail.com (E.X.-E.); 2 Unitat de Genètica, Departament de Ciències Fisiològiques, Genes Disease and Therapy Program IDIBELL-Institute of Neurosciences, Universitat de Barcelona, L’Hospitalet de Llobregat, 08907 Barcelona, Spain; ekaitz_errasti@ub.edu; 3 Centro de Investigación en Red de Enfermedades Raras (CIBERER), ISCIII, 28029 Madrid, Spain; 4 Institute of Biochemistry and Cell Biology, Italian National Research Council (CNR), I-00015 Rome, Italy; daniela.marazziti@cnr.it

**Keywords:** megalencephalic leukoencephalopathy with subcortical cysts, GlialCAM, MLC1, GPRC5B, GPR37L1, GPR37, glia, ion, water homeostasis

## Abstract

Megalencephalic leukoencephalopathy with subcortical cysts (MLC) is a rare type of vacuolating leukodystrophy (white matter disorder), which is mainly caused by defects in MLC1 or glial cell adhesion molecule (GlialCAM) proteins. In addition, autoantibodies to GlialCAM are involved in the pathology of multiple sclerosis. *MLC1* and *GLIALCAM* genes encode for membrane proteins of unknown function, which has been linked to the regulation of different ion channels and transporters, such as the chloride channel VRAC (volume regulated anion channel), ClC-2 (chloride channel 2), and connexin 43 or the Na^+^/K^+^-ATPase pump. However, the mechanisms by which MLC proteins regulate these ion channels and transporters, as well as the exact function of MLC proteins remain obscure. It has been suggested that MLC proteins might regulate signalling pathways, but the mechanisms involved are, at present, unknown. With the aim of answering these questions, we have recently described the brain GlialCAM interactome. Within the identified proteins, we could validate the interaction with several G protein-coupled receptors (GPCRs), including the orphan GPRC5B and the proposed prosaposin receptors GPR37L1 and GPR37. In this review, we summarize new aspects of the pathophysiology of MLC disease and key aspects of the interaction between GPR37 receptors and MLC proteins.

## 1. Introduction

Megalencephalic leukoencephalopathy with subcortical cysts (MLC) is a rare genetic type of leukodystrophy (OMIM 604004). It is a childhood-onset hereditary disease characterized by white matter vacuolation and macrocephaly, which is developed during the first year of life. Most MLC patients present a progressive loss of motor functions with ataxia and spasticity, cognitive decline and epileptic seizures [1]. Symptomatology often worsens after fever or mild head trauma [2].

Magnetic Resonance Imaging (MRI), which is used for diagnostics, shows that patients display diffuse signal abnormality and swelling of the cerebral white matter together with the presence of subcortical cysts, mainly in the anterior temporal regions [3]. Furthermore, MRI together with the histopathology of the brain of an MLC patient revealed that myelin contains water-filled vacuoles [4]. Up to date, MLC has no cure. Treatment is symptomatic in combination with supportive care.

Two different phenotypes of MLC disease have been described: a classical and a remitting phenotype [5]. The classical phenotype is the most commonly found in patients. It is caused by autosomal recessive mutations in either the *MLC1* or the hepatic cell adhesion molecule (*HEPACAM*) genes, resulting in two disease subtypes, namely MLC1 or MLC2A. The remitting phenotype, named MLC2B, is caused by dominant mutations in *HEPACAM* [6]. Approximately 76% of patients present mutations in *MLC1*, 22% in *HEPACAM* and 2% of MLC cases cannot be explained by mutations in these two genes, suggesting that others might be implied in the disease [2].

*MLC1* encodes for a membrane protein with eight predicted transmembrane domains whose function remains unknown. It is expressed exclusively in the brain in astrocytes surrounding blood vessels and Bergmann glia in the cerebellum [7]. More than 50 mutations have been described for *MLC1*, including missense, deletions, insertions and nonsense mutations [1].

On the other hand, *HEPACAM* encodes for a cell adhesion molecule of the immunoglobulin (Ig) family named GlialCAM, which is expressed predominantly in neurons, astrocytes and oligodendrocytes [6]. GlialCAM was first identified in hepatic cancer, where it was downregulated, but it is predominantly expressed in glial cells [8]. GlialCAM can form interactions with other GlialCAM molecules in *cis* (within the same cell) or *trans* (between different cells) [9]. GlialCAM acts as an endoplasmic reticulum (ER) chaperone for MLC1 [10] and it also helps MLC1 to reach astrocyte-astrocyte junctions where both proteins co-localize [6,11]. Apart for being involved in MLC, recently, it has been described that autoantibodies recognizing GlialCAM might be involved in the development of multiple sclerosis [12].

The pathophysiological mechanisms leading to MLC are still unclear [13]. Even though the function of the MLC1/GlialCAM complex is unknown, it has been hypothesized that it may have a role in the regulation of ion/water homeostasis, as it interacts with different transporters and ion channels. Thus, it has been shown that the complex interacts directly with the chloride channel 2 (ClC-2) [14], the gap junction alpha 1 protein (connexin 43, Cx43) [15,16,17], Na^+^/K^+^-ATPase [18,19], and it is thought to regulate indirectly the activity of volume-regulated anion channel (VRAC) [10,20,21] or the calcium-permeable channel TRPV4 [22]. Recent bioinformatics developments have suggested new potential functions for the GlialCAM/MLC1 complex (see Section 2).

## 2. Novel Insights into GlialCAM/MLC1 Function by Alphafold Structural Models

Powerful sequence-alignment methodologies have been used to identify possible functional MLC1 homologues. The underlying assumption of these sequence-based methods is that proteins with similar sequences adopt a similar fold. As protein folding determines function, the detection of evolutionary relationships between proteins can be used to predict functions of non-annotated protein sequences. Thus, MLC1 sequence identity analysis by BLAST/PSI-BLAST algorithm identified the voltage gated potassium channel Kv1.1 alpha subunit (KCNA1) as the protein with the highest sequence identity (less than 20% amino acid identity) [23], suggesting that MLC1 could act as an ion channel. In agreement with this hypothesis, it is known that MLC1 can form homo-oligomers, a characteristic found in many ion channels proteins [24]. Moreover, MLC patients may present epileptic seizures, which is a common feature in ion channel diseases, but not in leukodystrophies [25]. Taking all these data into consideration, the first hypothesis regarding MLC1 function was that it could act as an ion channel. Nevertheless, voltage-clamp measurements in *Xenopus* oocytes did not detect any changes in the conductance and neither did patch clamp measurements in HEK293T or HeLa transfected cells. For those experiments, different pulse protocols and various pulse durations were applied and no conductivity of MLC1 was detected, even in the presence of GlialCAM [26].

**Figure 1:**
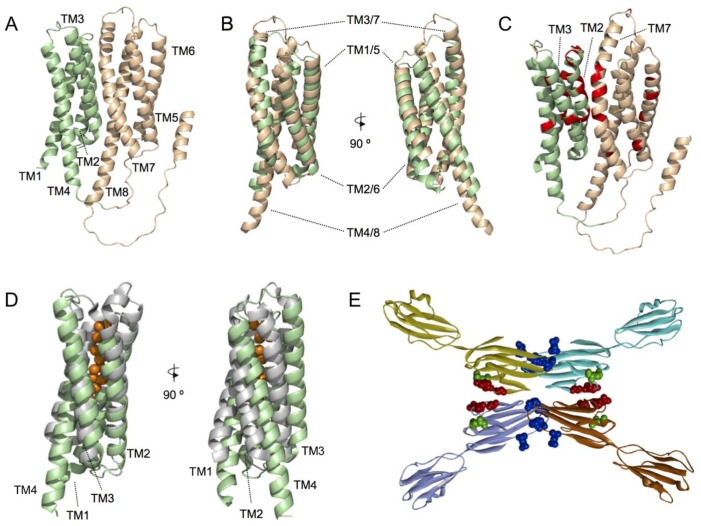
**Structural models of MLC1 and GlialCAM proteins.** (**A**) Alphafold structural model of monomeric MLC1. Helices 1 to 4 and 5 to 8 are coloured pale green and wheat, respectively. (**B**) Structural superimposition of the two MLC1 subdomains, comprised of TMs 1 to 4 (pale green) and 5 to 8 (wheat), respectively. Two different views, rotated by 90°, are shown. (**C**) MLC causing mutations (red) depicted in the MLC1 structural model. Preferential localization of mutated residues is located in the interaction surface defined by the two MLC1 subdomains (TMs 1 to 4 (pale green) and 5 to 8 (wheat)). (**D**) Structural superimposition of MLC1 structural model TM1 to 4 (pale green) and a prokaryotic soluble copper storage protein from *Streptomyces lividans* (light grey) (PDB ID 6Q6B). Two different views, rotated by 90°, are shown. (**E**) Summary of the structural model proposed for GlialCAM homodimers forming *cis* and *trans* interaction through different surfaces of its IgV domain. *Cis* dimerization is achieved by interactions between two opposing beta-strands of the IgV domain and *trans* interactions occur between salient loops of both IgV domains. Residues mutated in MLC2A patients (recessive) are shown in green, Residues mutated in MLC2B patients are shown in red or blue (affecting blue the interactions in *trans* only).

The functional characterization of MLC1 is necessary to understand its physiological relevance. As the 3D structure of a protein is believed to be responsible for its biological function, protein structure can provide a better insight into which protein fragments contribute most to the functionality of a protein compared to the primary sequence. In fact, residues located far apart in primary sequence may be close in the 3D structure. Recently reported MLC1 structural models [27,28] suggest a protein fold based on a 4 + 4 structural repeat, involving transmembrane regions (TMs) 1–4 and 5–8 (Figure 1A,B). Interestingly, although mutations found in MLC patients are spread through all the protein sequence, a significant number of identified missense MLC1 mutations are mainly localized on the interphase between MLC1 internal repeats (Figure 1C). As MLC1 mutations have been reported to cause protein instability, which consequently causes its degradation at the endoplasmic reticulum or lysosomes [29,30,31], we propose that proper interaction between MLC1 internal repeats is of key relevance for proper protein folding and endoplasmic reticulum sorting. Similarly, recently reported MLC1 homo-trimeric complex in detergent micelles and proteoliposomes [24], suggests that mutations affecting MLC1 monomers interaction would also affect protein stability. However, the lack of a 3D structure at atomic resolution precludes the identification of putative protein-protein interaction regions.

The growing number of known protein structures and structural models has motivated the interest into developing approaches that aim to identify the protein functions from the structure. Among them, methods comparing the global fold of a query protein with other template proteins of known function are commonly used. Identification of proteins similarly folded to whole MLC1 and TM1-4 MLC1, using the PDBeFold algorithm [32], revealed both prokaryotic and eukaryotic proteins with a similar 3D fold. Particularly, PDBeFold analysis of the TM1-4 MLC1 subdomain resulted in the identification of a prokaryotic soluble copper storage protein from *Streptomyces lividans* (PDB ID 6Q6B) (Figure 1D), suggesting that maybe MLC1 would be involved in ion sensing. However, further research should be conducted to solve this issue.

In addition, based on the identification of the interactome of GlialCAM and MLC1, we recently proposed that MLC1 could act as a tetraspanin-like molecule [16]. Tetraspanins are cell-surface proteins with four transmembrane domains, which can homo- and hetero-oligomerize. They participate in a wide variety of cellular processes such as adhesion, differentiation and cell activation [33]. In many cases, they also form a tight complex with proteins belonging to the Ig superfamily, as it happens with MLC1 and GlialCAM.

Our recent work based on 3D models combined with biochemical analyses indicated that GlialCAM forms interactions in *cis* through an interaction surface comprising residues Glutamate 86 to Arginine 92 in the IgV domain [9] (Figure 1E). Furthermore, two other loops might also be involved in the formation of *trans*- interactions, which are blocked by a nanobody generated against GlialCAM. Residues causing dominant MLC are located in these two interaction surfaces (Figure 1E). It remains to be determined whether the IgC2 domain contributes to the formation of lateral interactions as it has been observed in other Ig proteins, or if it also mediates interaction with the MLC1 protein.

## 3. Physiological Processes Affected in MLC Deduced from Animal Models of MLC

The main characteristic observed in MLC patients is that the brain is enlarged with increased water accumulation. Water is accumulated in subcortical cysts but also, in a general manner, in the outer part of myelin [4] and in astrocytes surrounding blood vessels [34]. Similarly, the lack of MLC1 or GlialCAM in knockout (KO) mice [18,35,36,37] or KO zebrafish [7,38] leads to an increased brain water content and cerebellar white matter vacuolation, although no cysts are observed. Moreover, in humans, the oedema is localized mainly in the subcortical white matter, while vacuoles are found in the cerebellum of KO mice. Although vacuoles of water in myelin sheaths that enwrap axons of central neurons can be observed in both patient biopsies and mice samples, still the developmental myelination process is not altered. Considering astrocytes, histological studies revealed that *Mlc1* KO mice presented abnormal astrocytes with thicker cell processes, swollen cell bodies and enlarged end-feet, although the length and number of these cells were not altered [34]. Primary astrocytes from *Mlc1* KO showed an increase in the number of vacuoles [7]. In summary, although there are differences between human and animal models, they show in common an increase in the content of water in the brain, which is accumulated in the form of vacuoles in myelin or in astrocytes.

Why does the lack of MLC1 lead to an increased water content in the brain? The first hypothesis was that MLC1 functions as a water or ion channel, and its defect causes water accumulation due to osmotic alterations linked to neuronal activity [23]. However, this activity has not been detected after expressing the MLC1 protein alone or together with GlialCAM in different cell systems [26]. Thus, a search for other proteins interacting with MLC1 and/or GlialCAM to find a connection with the patient’s brain phenotype was developed. Unsurprisingly, several transporters and ion channels were identified by different groups (for review [13]). In an extensive proteomic analysis [16], we have recently identified the following interactor proteins: the chloride channel ClC-2, the gap junction protein Cx43, the glutamate transporter EAAT1/2, the alpha2 and beta2 subunits of the sodium/potassium ATPase, the sodium bicarbonate transporter NBCe1, the glucose transporter GLUT1, and the sodium calcium exchanger. Furthermore, although not appearing as MLC interacting proteins, other activities have been shown to be affected by the lack of MLC1 such as the volume regulated anion channel (VRAC) or the calcium-permeable channel TRPV4. It has been clearly demonstrated that the activity of the chloride channels ClC-2 [37] and VRAC [21,35] and of the gap junction protein Cx43 [15] are affected in vivo. Thus, one possible hypothesis is that the lack of activity of some of these proteins is the reason that explains myelin vacuolization. In agreement with these hypotheses, a *Clcn2* KO mouse model displayed widespread vacuolization, including the cerebellum [37]. Similarly, defects of connexins have also been linked to myelin vacuolization [39]. As LRRC8 proteins of VRAC channels are involved in cell volume regulation and their absence cause cell vacuolation in several tissues, it will be important to analyse whether a conditional (because the complete KO is deleterious [40]) astrocyte KO of LRRC8A [41] (the main constituent of the VRAC channel [42,43]) also displays astrocyte and myelin vacuolization.

In contrast to other leukodystrophies, MLC patients can suffer from seizures [25]; still the underlying mechanism that links MLC to epilepsy is not known. In this sense, both *Glialcam* and *Mlc1* KO mice present an abnormal extracellular K^+^ dynamics and neuronal network activity, as they had an epileptiform brain activity and a lowered seizure threshold [44]. As described previously, defects in either the MLC1 or GlialCAM interacting proteins’ function might also affect extracellular potassium dynamics. Astrocyte uptake is mainly mediated by the Na^+^/K^+^ ATPase pump [45], an interactor of MLC1 [16,18,19]. In *Mlc1* KO mice, there is a reduced expression of the inward rectifier potassium channel Kir4.1 involved in potassium clearance [35], whose absence is known to lead to hyperexcitability and epilepsy [46]. The interaction of GlialCAM with the ClC-2 chloride channel increases total current amplitudes and abolishes the rectification of ClC-2, which is thus opened at positive voltages [14]. In addition, this interaction opens the ClC-2 common gate, which is closed by acidic pH. This reduction in the inward rectification will allow the influx of chloride, which may be needed to maintain the electroneutrality after potassium intake in glial cells [47]. Connexins are also essential to disperse local high potassium concentrations through a glial syncytium. It must be considered that disturbed astrocyte regulation of water homeostasis in MLC affecting the VRAC channel might also cause hyperexcitability of neuronal networks and seizures.

Importantly, perivascular astrocytes by itself, where MLC1 expression is higher and can even be considered a marker of these cells [48], are essential to the maintenance of an adequate blood brain barrier, which is also important for the clearance of potassium [49]. In this sense, it has recently been shown that the lack of MLC1 affects the perivascular astrocytic processes’ molecular maturation and organization. In *Mlc1* KO mice, an accumulation of fluid in the brain occurs, although this does not alter the blood-brain barrier integrity and neither the organization of the endothelial network. It has been determined that MLC1 might play a role in contractile maturation of vascular smooth muscle cells, arterial perfusion, and neurovascular coupling. Its absence disturbs the postnatal acquisition of contractile properties by vascular smooth muscle cells and disrupts blood perfusion, vessel diameter and neurovascular coupling [50]. Thus, not only affecting different ion channels and transporters in astrocytes can cause water increase and it might be that disruption of gliovascular unit by itself might contribute also to the increase in water content and seizures observed in MLC patients.

Another important aspect to consider in the pathophysiology of MLC is that MLC1 is expressed in astrocytes, whereas its auxiliary subunit GlialCAM is expressed in astrocytes, oligodendrocytes, and neurons [15,37]. Importantly, GlialCAM regulates astrocyte competition for territory and morphological complexity in the developing mouse cortex [15]. It has been shown that the lack of MLC1 causes GlialCAM mislocalization by an unknown mechanism in astrocytes [7], but also in oligodendrocytes [37], possibly because astrocytic GlialCAM interacts in *trans* through the IgV domain with oligodendrocytic GlialCAM. Moreover, the mislocalization of GlialCAM also affect to the localization of ClC-2 in oligodendrocytes, as shown by immunofluorescence and electrophysiological measurements of ClC-2 activity in oligodendrocytes on cerebellar slices [37]. In line with these studies, it has been recently shown that the lack of GlialCAM in astrocytes in vivo decreases synaptic inhibition and, therefore, increases excitation, which may also explain seizures [15]. As ClC-2 and GlialCAM are also expressed in inhibitory synapses, this change in synaptic function might also be related to disorganization of GlialCAM (astrocyte)-GlialCAM (neuron) trans contacts. It could also be related to the mislocalization of other interacting partners such as Cx43, which in turn might also interact with connexins present in oligodendrocytes (connexin 47) [51] and/or neurons.

As previously stated in this review, the functional role of the GlialCAM/MLC1 complex is still unknown. Nevertheless, it has been described that several proteins and processes related to brain homeostasis are affected in a GlialCAM or MLC1-dependent manner and it is not clear how GlialCAM and MLC1 exert this effect on the activity of different ion channels and transporters. A suggested hypothesis argues that they might influence signalling cascades by yet undefined mechanisms [52], which may regulate these channels or transporters. In this regard, GlialCAM and MLC1 have been related to signal transduction changes. For instance, it has been described that the overexpression of human MLC1 in astrocytes decreases the phosphorylation of extracellular signal-regulated kinases (ERK), whereas primary astrocytes lacking MLC1 show an increase in phosphorylation [21]. Nonetheless, the mechanisms involved in this process remain unresolved.

**Figure 2:**
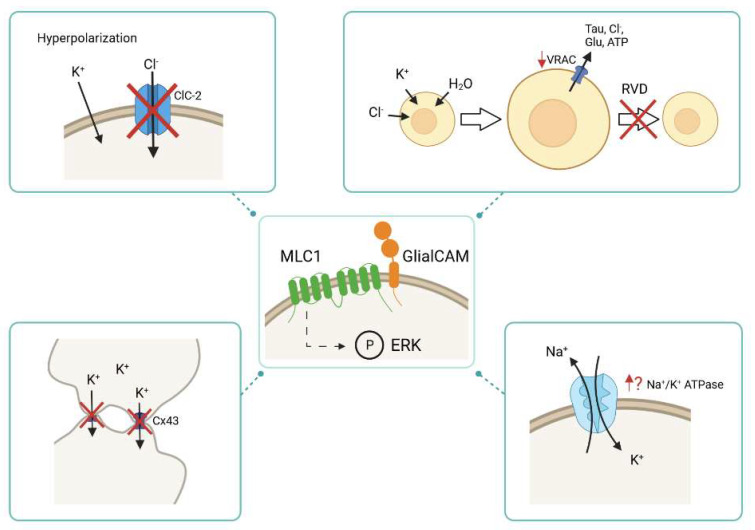
**Physiological alterations caused by the lack of MLC1 and/or GlialCAM.** MLC1/GlialCAM might regulate the activity of different transporters and ion channels through phosphorylation of ERK signalling transduction cascade. In the absence of MLC proteins, the activity of different transporters and ion channels is altered, such as the activity of ClC-2, VRAC, Cx43 and Na^+^/K^+^ ATPase. During the hyperpolarization phase of a neuronal action potential, the activity of the chloride channel ClC-2 is decreased in the absence of MLC1/GlialCAM. The lack of MLC proteins diminishes VRAC activity, which leads to an impaired regulatory volume decrease (RVD) response: an important mechanism to shrink cells after cell swelling. The Cx43 localization in the absence of MLC1/GlialCAM is affected as it is internalized and it is no longer located at cell-cell junctions. It is thought that the activity of the Na^+^/K^+^ ATPase is altered when MLC proteins are not present.

In summary, GlialCAM and MLC1 seem to regulate the activity of many different transporters and ion channels in different cell types (Figure 2), possibly by regulating phosphorylation of these proteins. An anomalous activity of these proteins might contribute to the defects observed in the regulation of the extracellular water and ionic homeostasis, which could explain the increased water content and seizure susceptibility of MLC patients.

## 4. Regulation Mechanism of Different Transporters and Ion Channels by MLC Proteins: Is a GPCR Link the Answer?

Recently, our research group identified the GlialCAM interactome through an approach based on affinity purifications (APs) [16]. Four different antibodies specific for GlialCAM were used on samples consisting of membrane fractions prepared from whole brains from adult animals. These included wild type (WT) rats and mice, as well as *Glialcam* KO mice. The previously validated interactors MLC1, ClC-2 and GlialCAM itself were retained in all APs with high efficiency, reinforcing the robustness of this approach. We could identify as many as 21 proteins within the GlialCAM interactome in the rodent brain, some of them already linked to MLC as mentioned above. Within the proteins identified as part of the network of GlialCAM, there were three specific G protein-coupled receptors (GPCRs). Specifically, we retrieved the orphan receptors GPRC5B [53], which we suggested that mutations in this gene could be found in MLC patients without mutations in *MLC1* and *HEPACAM*, and the proposed binders of prosaposin GPR37 and GPR37L1 [54,55]. It is interesting to note that the latter two GPCRs belong to the same protein family. Therefore, we proceeded to determine the potential interaction and the relationship between these GPCRs and MLC-related proteins (GlialCAM/MLC1 and ClC-2). As the purpose of this review is to update the knowledge of the GPR37 family, we will focus on these two proteins.

The GPR37 and GPR37L1 proteins are part of class A rhodopsin-like family of GPCRs, which comprises 80% of all identified GPCRs [56]. Both are considered orphan receptors as no ligand has conclusively been linked to them in vitro [55]. The two GPCRs are widely expressed in the CNS [57]. GPR37 is mainly expressed in the cerebellum, corpus callosum, medulla, putamen, caudate nucleus, substantia nigra and the hippocampus [58,59,60,61]. Specifically, oligodendrocytes are the cell type displaying a higher expression of GPR37 together with certain subsets of neurons like dopaminergic neurons in the substantia nigra [62]. On the other hand, GPR37L1 is exclusively expressed in glial cells within the brain, in particular Bergmann glia astrocytes in the cerebellum [63], as well as immature oligodendrocytes [64].

Studies carried out in *Gpr37* KO mice revealed that GPR37 acts as a negative regulator of oligodendrocyte differentiation and myelination. The lack of GPR37 leads to premature differentiation of pre-myelinating oligodendrocytes to myelin producing cells. This alteration is a cause of central nervous system hypermyelination in these mice, already at a very young age and up until adult stages [64]. Furthermore, experiments performed both in primary oligodendrocytes and in brain-derived samples obtained from *Gpr37* KO mice show an increase in ERK1/2 phosphorylation. Pharmacological inhibition of MEK1/2 and ERK1/2 seemed to stop premature differentiation of oligodendrocytes in the absence of GPR37. This inhibition, however, did not affect normal cell proliferation [65]. In addition, adenylyl cyclase inhibition resulted in the impairment of ERK1/2 translocation to the nucleus. Taken together, these data suggested that this pathway is indeed responsible for GPR37 activation during oligodendrocyte differentiation.

Regarding GPR37L1, several studies have highlighted its relevance in the developing brain. One study showed that the lack of Gpr37l1 led to altered cerebellar development in mice [63]. The animals displayed a reduction in neuronal granule cell precursors together with premature maturation of Bergmann glia and Purkinje neurons. Cerebellar layer formation was also altered. However, the authors observed that KO mice seemed to perform better in motor tasks. Motor learning was improved both in juvenile and adult stages, while the adult animals also showed better coordination skills. Furthermore, it has been suggested that Gpr37l1 could play a role in recovery after ischemic injuries, possibly by modulating glutamate transporters [66], which also form part of the GlialCAM interactome [16].

## 5. Biochemical Relationship between the GPR37/GPR37L1 and MLC Proteins

**Figure 3:**
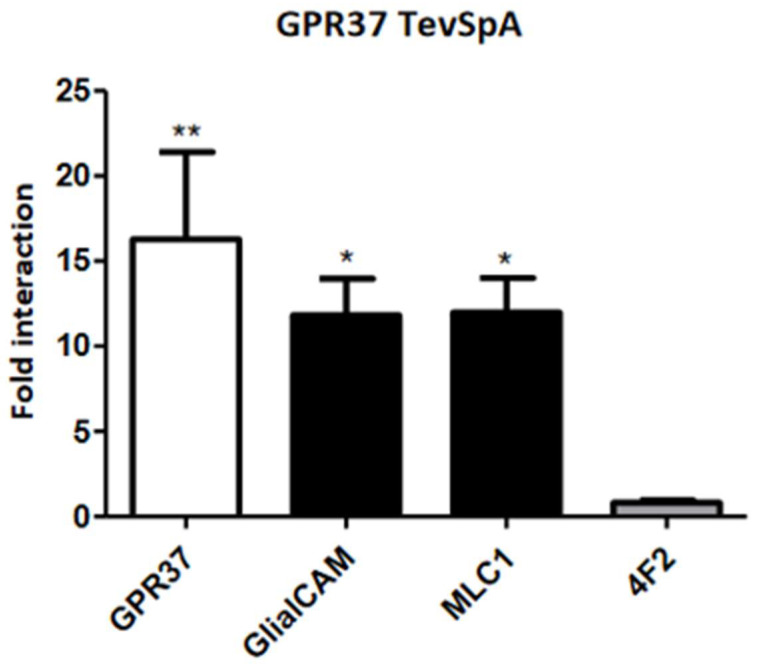
**Direct Interaction between GPR37 and GlialCAM/MLC1.** Results of split-TEV interaction assays in HeLa cell using GPR37 C-terminally tagged with the N-terminal part of the TEV protease (TevSpA). They were co-transfected with different constructs (GPR37, GlialCAM, MLC1 and 4F2 as a negative control) C-terminally tagged with the C-terminal part of the TEV protease. Plotted data combine the results from three independent experiments. Statistical significance was obtained comparing the interaction within each group to the interaction with the negative control in Bonferroni’s multiple comparison test (* *p* < 0.05, ** *p* < 0.01 in the test versus 4F2). Results show that GPR37 is able to homo-oligomerize and hetero-oligomerize with GlialCAM and MLC1, in the same manner as shown for GPR37L1 by BRET.

As detailed above, these two GPCRs, that were identified as members of the GlialCAM interactome, play an important role in mediating a variety of processes in the central nervous system. Hence, our research group was particularly interested in the analysis of the potential role of these GPCRs in MLC pathophysiology. The first experiments that were carried out aimed to validate the proteomics data regarding the interaction between these proteins and GlialCAM/MLC1 [16]. As MLC1 and GPR37L1 are both expressed in astrocytes, we initially focused our studies on this GPCR. We could establish that there was co-localization between GPR37L1 and MLC1 by immunofluorescence in mouse primary astrocytes. Furthermore, both proteins were in proximity in the same cells, as assessed by Proximity Ligation Assay (PLA) [16]. Moreover, the ability of each of the two GPCRs to directly interact with either MLC1 or GlialCAM was monitored by split-tobacco etch virus (TEV) assays for GPR37 (Figure 3) and bioluminescence resonance energy transfer (BRET) studies in HEK293T cells for GPR37L1 [16].

The above summarized results support the formation of complexes between the GPCRs and MLC-related proteins in living cells. More work was carried out to further characterize the nature of the relationship between these proteins and the physiological role of these complexes. In collaboration with the group led by Daniela Marazziti, we started to address these questions using the *Gpr37l1* constitutive KO mice [63]. As the animals showed no alteration of adult cerebellar layer cytoanatomy and organization and there was no apparent sign of gliosis, it was considered that the analysis of MLC proteins in *Gpr37l1* KO mice could elucidate direct effects of GPR37L1 on MLC protein expression and function.

Biochemically, the first step was to analyse the consequences of the lack of GPR37L1 on MLC1 and GlialCAM protein levels [16]. Western blot experiments of cerebellar membrane fractions indicated that both proteins were upregulated in the *Gpr37l1* KO samples. Likewise, ClC-2 protein levels were also increased. Similarly, in immunofluorescence labelling experiments of MLC1 and GlialCAM in cerebellar Bergmann glia samples, an increased signal was observed. These in tissue results were consistent with what was observed in primary astrocytes derived from these mice, in which an increased signal for MLC-related proteins was revealed. However, the MLC proteins and ClC-2 showed a more dotted pattern compared to the WT signal. To determine whether MLC1 subcellular localization was altered in *Gpr37l1* KO mice, we proceeded to detect MLC1 by electron microscopy (EM) immunogold experiments. These experiments showed that the localization of MLC1 in Bergmann glia or in perivascular astrocytic processes was not affected. In summary, we concluded that the lack of GPR37L1 in mice upregulates MLC protein levels without altering their localization [16]. No studies have so far been performed in *Gpr37* KO mice.

## 6. Possible Role of GPR37 and GPR37L1 in MLC Pathophysiology

**Figure 4:**
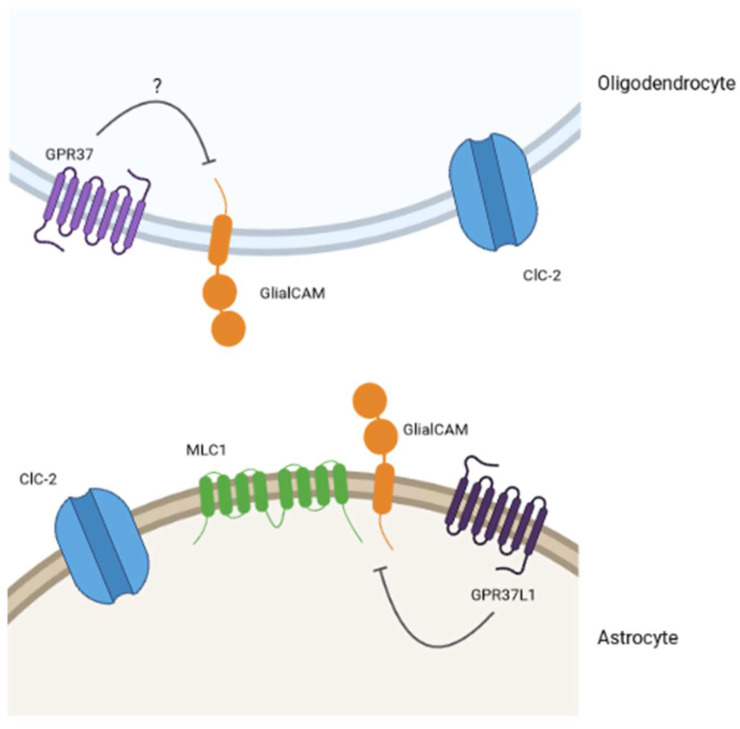
Model for the interplay between GPCRs and MLC-related proteins. The existing pieces of evidence in the literature and the results from our lab lead us to think that GPR37 and GPR37L1 would be part of an interplay with MLC-related proteins. Specifically, we hypothesize (question mark for GPR37 in oligodendrocytes) that these GPCRs would be negative regulators of the physiological MLC1/GlialCAM complex activity.

As the lack of GPR37L1 increases MLC proteins levels, it was suggested that GPR37L1 might be a negative regulator of MLC proteins in astrocytes (Figure 4). Interestingly, previous studies [67] have suggested that GPR37 interacts and negatively regulates dopamine transporters by regulating their endocytosis and trafficking. As stated previously, GPR37 has been described to act as a negative regulator of myelin formation [64]. As the proper expression of GlialCAM/MLC1 is necessary for myelin homeostasis, we could hypothesize that GPR37L1 would exert a similar negative effect regulating the GlialCAM/MLC1 complex during development (Figure 4).

Previous studies indicated that the interaction between GlialCAM/MLC1 and ClC-2 in primary cultured astrocytes was dynamically regulated, and it was observed only in depolarizing conditions [47]. Then, we addressed whether the interaction between GPR37L1 and MLC1 was also regulated in a dynamic manner, and compared the interaction between GPR37L1 with MLC1 in physiological versus depolarizing conditions in primary astrocyte cultures [16]. The PLA assays indicated that the interaction between GPR37L1 and MLC1 was decreased, whereas the interaction with the orphan GPCR5B was increased. As GPRC5B interacts more with MLC1 in depolarizing and hypotonic conditions, the activity of GPRC5B might be important in metabolic processes related to changes in the ionic composition. In contrast, we hypothesize that signalling through GPR37L1 might be related to cell differentiation processes. In line with this hypothesis, as mentioned previously, the lack of GPR37L1 resulted in an increase of phospho-ERK1/2, which has also been seen in *Mlc1* KO cells [21].

Another similar correlation has been found recently between MLC proteins and GPR37L1. As mentioned previously, MLC patients [25] and *Mlc1* KO mice [44] show increased seizure susceptibility. A homozygous GPR37L1 variant (c.1047G > T [Lys349Asp]) has been found in a patient with myoclonus epilepsy [68]. Furthermore, both *Gpr37* and *Gpr37l1* KO mice showed an increase in seizure susceptibility [68]. These results are consistent with a possible functional link between these GPCRs and MLC disease. However, it is important to state that future research is needed to understand how GlialCAM and MLC1 modulate GPCR-associated signalling processes.

## Data Availability

Not applicable.
